# CSA declaration of next-generation reperfusion therapy for ischaemic stroke

**DOI:** 10.1136/svn-2024-003110

**Published:** 2024-01-17

**Authors:** Chinese Stroke Association

**Keywords:** Stroke, Thrombolytic Therapy, Thrombectomy, Thrombolysis, Technology

 Cerebrovascular disease is a significant health concern that threatens people’s health and life. About 80% of its burden comes from ischaemic stroke. Early reperfusion therapy is the key to timely restore blood flow to salvageable ischaemic brain tissue.

Reviewing the history of reperfusion therapy for ischaemic stroke, there have been two major breakthroughs: in 1995, following the publication of the National Institute of Neurological Disorders and Stroke (NINDS) rt-PA Stroke Study, thrombolytic therapy with intravenous alteplase significantly reduced disability and improved the quality of life for patients who had a stroke, marking the beginning of the era of reperfusion therapy for ischaemic stroke. Twenty years later, in 2015, several large clinical trials demonstrated that endovascular thrombectomy, in addition to intravenous thrombolysis with alteplase, further improved rate of recanalisation and reduced disability among patients who had a stroke. These new breakthrough treatment propelled reperfusion therapy into another new era.

In recent years, the establishment of organised stroke care systems has provided a strong momentum perfusion therapy for ischaemic stroke. The establishment of stroke centres at all levels and the application and promotion of mobile stroke units in our country have significantly reduced prehospital and in-hospital delays to treat patients who had a stroke, therefore improved the effectiveness of reperfusion therapy. Today, the phrase ‘Time is Brain’ has evolved from a slogan to actionable practices.

The application of artificial intelligence (AI) has greatly expanded the accessibility of reperfusion therapy for ischaemic stroke. With the help of AI tools such as ischaemic penumbra identification, the time window for reperfusion therapy has been extended to 24 hours, providing a greater opportunity for a broader range of patients to receive and benefit from the reperfusion therapy.

In 2021, the National Health Commission of China included ‘raising the rate of reperfusion therapy for acute ischaemic stroke’ as a goal of national healthcare quality and safety improvement. Subsequently, a series of initiatives such as the ‘Brain Care Action’, ‘Rapid Action’, and ‘Craftsmanship Action’ were implemented nationwide in order to achieve this goal.

Despite the initial increase in the rate of reperfusion therapy for acute ischaemic stroke due to these initiatives and the rate of intravenous thrombolysis reached 40% for eligible patients, the rate of endovascular thrombectomy to 7.1%, there is still substantial room for improvement. Raising the proportion and quality of reperfusion therapy for ischaemic stroke has become the most urgent task at the present.

Time has bestowed us this glorious but challenging responsibility to ensure that Chinese patients who had an ischaemic stroke receive the treatment consistent with the international standards.

To advance the ‘Healthy China Action - Implementation Plan for Cardiovascular and Cerebrovascular Disease Prevention and Control (2023-2030)’ effectively, we propose the following: mobilise all sectors of society and seize this rare historical opportunity to further enhance the quantity and quality of reperfusion therapy for ischaemic stroke. Fully implement new generation of thrombolytic drugs, endovascular retrieval devices, organised stroke care systems (such as emergency stroke units), artificial intelligence and smart healthcare technologies, as well as the national reperfusion registry platform. By 2030, all secondary and above-level medical institutions capable of treating cerebrovascular diseases should offer intravenous thrombolysis, raising the proportion of Chinese patients who had an ischaemic stroke receiving intravenous thrombolysis to 80%, the rate of endovascular thrombectomy to 30%, and safety of reperfusion therapy at the advanced level of world.

We will collectively advance and implement the following actions:

1. Accelerate the evidence-based evaluation of new generation of thrombolytic drugs (tenecteplase, reteplase, etc.), promote their approval for treating ischaemic stroke, and update the guidelines for reperfusion therapy. Encourage the development of novel thrombolytic agents to provide a wider range of drug options for the new era of reperfusion therapy.
2. Actively promote the dissemination and training of thrombectomy techniques, encourage the development of integrated imaging evaluation systems and new thrombectomy devices, and conduct evidence-based medical research on thrombectomy. Advance the establishment of thrombectomy demonstration centres and workstations to make arterial reperfusion therapy more widely accessible, efficient and safe.
3. Strengthen the construction of ‘1-hour stroke rescue circle’ and stroke centres, establish a more efficient and China-specific organised stroke medical care system, and reduce prehospital and in-hospital delays in reperfusion therapy through technological development and innovative models. Actively promote the emergency stroke unit model based on low-field strength MRI, advance evidence-based medical research and continue quality improvement in emergency stroke units to provide a Chinese solution of constructing an international organised stroke medical care system.
4. Actively promote the research and application of artificial intelligence and intelligent medical technologies, including salvageable brain tissue identification and decision-support systems, to expand the indications for reperfusion therapy to benefit more patients who had an ischaemic stroke.
5. Encourage basic, translational and clinical research combining with brain cell protection and reperfusion therapy so to expand the time window for reperfusion therapy and improving its effectiveness.
6. Conduct national registration and safety monitoring of ischaemic stroke reperfusion therapy to provide a data foundation for the standardised advancement and real-world research of reperfusion therapy.
7. Establish new training and demonstration centres for the next generation of reperfusion therapy and technology and provide solid support for the training of new talent to provide reperfusion therapy.
8. Establish partnerships between medical institutions and non-profit organisations, continue to enhance public awareness and education on the recognition and medical knowledge of acute stroke and reperfusion therapy, emphasise the need for early treatment in the acute phase of stroke, especially for ischaemic stroke, and highlight the importance of timely application of reperfusion therapy to improve patients’ chances of recovery.
9. Advocate for clinical research related to reperfusion therapy to provide more Chinese evidence to international evidence-based medicine on reperfusion therapy.

We hope, through the collaborations of all sectors of our society, a new era of reperfusion therapy for ischaemic stroke will begin.

